# Assessment of a Social Media‐Based Method for Determining Raptor Diet

**DOI:** 10.1002/ece3.71415

**Published:** 2025-06-04

**Authors:** Leo Biggs, Greg S. Baxter, Stephen J. S. Debus, Neal Finch, Anysha Riggs, Hayden Houweling, Melissa Appleby, Peter J. Murray

**Affiliations:** ^1^ School of Agriculture and Environmental Science University of Southern Queensland Toowoomba Queensland Australia; ^2^ Zoology, School of Environmental and Rural Science University of New England Armidale New South Wales Australia

**Keywords:** Bird of prey, citizen science, diet, method efficiency, predator, prey, raptor, social media

## Abstract

Accurate dietary information is important to improve ecological knowledge and assist in the conservation of threatened predators and their prey. Globally, many raptor populations are threatened and would benefit from accurate diet information. However, the existing methods of collecting diets can be time consuming, biased, or unfeasible for large spatial areas. Use of citizen science has been suggested to address these issues, but critical analysis of the method and comparison with existing methods is largely incomplete. Here, we evaluate the accuracy, efficiency, and biases of raptor diets data mined from social media. Two Australian coastal raptor species, Eastern Osprey ( *Pandion haliaetus* *cristatus*) and White‐bellied Sea‐Eagles (WBSE) ( *Haliaeetus leucogaster* ), were searched on Facebook and Instagram. Photographs and videos showing these raptors in possession of prey items were catalogued to form diets. The time taken to collect and identify prey items was recorded. The social media method was effective, producing large quantities of high quality media that recorded raptors and their prey. Study analyses utilised 1484 Eastern Osprey and 501 WBSE prey items posted between 2019 and 2023. Diet completeness was high, with a greater number of prey species observed in each region than previous studies. The prey identification rate to the family level was comparable with other direct visual observation methods at 58%. A consistent diet was observed between social media platforms. Eastern Osprey diet was similar to previous studies in New South Wales, except for a single prey family. WBSE diet was more varied than previous studies, which appeared biased by a locally high availability of certain prey species. The social media method was also efficient, with prey items collected and identified at a rate of 0.21 h per prey item; over 25 times faster than other raptor diets collected using direct visual observations. Furthermore, the social media method countered the biases of alternative methods such as small population samples, restricted spatial coverage, and over or under‐representation of animal classes. However, it produced new biases requiring quantification such as human activity hotspots, potential difficulty identifying small prey, and an over‐representation of easily identifiable or ‘interesting’ prey. Overall, the use of social media was effective and efficient at recording raptor diet and is encouraged for other raptors and predators.

## Graphical abstract

This study mined social media as a citizen science method to reduce the time burden and biases associated with recording accurate diet information for predators. In total, 1985 prey observations were recorded of two Australian coastal raptors and were used to produce diet profiles that were compared by region, social media platform, and against other diet collection methods. This social media method was successful and efficient at recording raptor diet, with an efficiency over 25 times faster than direct visual observations.

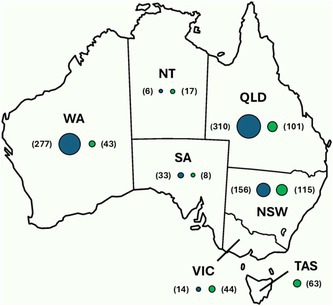

## 1 Introduction

Accurate diets are an important component of wildlife management, informing conservation of animal populations (Fryxell et al. 2014). Having a clear knowledge of diet is key to understanding prey and their habitat, helping to sustain populations of threatened predator species (Lortie et al. 2023). Raptors, members of the Accipitriformes, Falconiformes, Strigiformes, Cathartiformes, and Cariamiformes (McClure et al. 2019), are a group of avian predators that include many threatened species. Globally, 52% of raptor species have declining populations, and 18% are at threat of extinction (McClure et al. 2018). Therefore, over 290 of 557 raptor species would benefit from accurate diet information. However, recording accurate raptor dietary information is difficult and time consuming (Marti et al. 2007), resulting in a barrier to complete this work.

Traditional methods for determining raptor diet include pellet and prey remains collection from nest sites, and visual observations either in person or through remote cameras (Marti et al. 2007). The method utilised depends on the requirements for monitoring each raptor species, and sometimes more than one method is required to produce an accurate diet assessment (Marti et al. 2007; Mersmann et al. 1992). Comparisons between these methods demonstrate that diets formed from visual observations are the most complete, whereas diets composed from remains underestimate small prey items that can be consumed whole, and diets from pellets underestimate birds which are often plucked prior to consumption (Lewis, Fuller, et al. 2004; Margalida et al. 2007). Quantifying prey items using pellets can also be problematic, with the same prey item appearing in multiple pellets (Mersmann et al. 1992). However, despite providing more complete diets, visual observations are especially time consuming, but can be made more efficient using remote cameras (Lewis, DeSimone, et al. 2004).

Examples of novel methods used for recording raptor diet include stable isotope analyses (Baino et al. 2022; Resano‐Mayor et al. 2014) and DNA metabarcoding (Bourbour et al. 2019; Hacker et al. 2021). Stable isotope analyses can determine diet composition from different trophic levels and prey habitat (Tauler‐Ametller et al. 2018). However, it requires some prior knowledge of the predator's diet (Johnson et al. 2020) and cannot identify individual prey species (Resano et al. 2011). DNA metabarcoding compares sections or ‘barcodes’ of genetically sequenced prey items found in biological samples (e.g., faeces or pellets) to reference databases (Hebert et al. 2003). This method can provide good species‐level identification, but due to severe primer bias, cannot determine all taxa in a sample and subsequently cannot provide accurate biomass estimates (Elbrecht and Leese 2017). As such, there is no ideal method of determining raptor diet.

Furthermore, raptors exhibit spatial and temporal variety in diet, and sampling should be carefully designed to capture this variance (McMeans et al. 2015). However, many raptor studies do not adequately capture this variance, as it is not time or cost effective to sample large spatial areas over long time periods using the methods previously described (Panter et al. 2024). In an attempt to improve efficiency, some raptor diet studies are synchronous with breeding studies (Dykstra et al. 2003; Schulze et al. 2000; Thorstrom and La Marca 2000), and many diet collection methods are focused on nest sites (Panter and Amar 2022). Unfortunately, this often exacerbates this sampling problem, resulting in sampling bias from small samples of populations and spatial areas, and a temporal bias from the reduced sampling period. Citizen science has been suggested as a tool to mitigate these sampling biases, while improving efficiency. Although it remains unclear how accurate it is compared to other diet collection methods.

Citizen science has been used to record raptor diet through data mining online platforms such as Google Images, citizen science databases such as iNaturalist, and social media. Google Images was mined to determine the physical characteristics of Barn Owls ( *Tyto alba* ) and Black Sparrowhawks ( *Accipiter melanoleucus* ) (Leighton et al. 2016) and the diet of Martial Eagles ( *Polemaetus bellicosus* ) (Naude et al. 2019). Citizen science databases were used to determine the diet of caracaras (Panter et al. 2024; Pantoja‐Maggi et al. 2024). Multi‐platform methods were used to investigate sex, age, and temporal differences in the diet of Eurasian Sparrowhawks ( *Accipiter nisus* ) (Panter and Amar 2021, 2022), the diet of Montagu's Harriers ( *Circus pygargus* ) wintering in India (Kannan et al. 2023), and diet specialisations of Tiny Hawks ( *Accipiter superciliosus* ) (Berryman and Kirwan 2021). Although these methods appear to successfully produce diets, only the study by Kannan et al. (2023) compared diet completeness to an existing collection method (pellets). In addition, none of the studies investigated time efficiency for collecting diets. Furthermore, although some biases have been discussed, none of the papers provide a comprehensive review of biases, and the only bias that has been quantified is prey size in Panter and Amar (2021). The primary objectives of this study are to investigate the accuracy, efficiency, and biases of using citizen science, in particular social media, to record raptor diets. Accuracy in this study is defined as the completeness of the diets produced and to what taxonomic level prey can be identified. Efficiency is the time taken to record and identify each prey item.

Social media represents the largest potential source of data for citizen scientists, with billions of active users of the most popular platforms such as Facebook, Instagram, and Youtube (Dixon 2024). These platforms provide an easy‐to‐use framework for users to share digital content they have recorded. Globally, 4.7 billion photos are produced and stored each day (Nicholls 2023), recording the everyday lives of humans and their environment. Although the majority of photographs and videos are not uploaded to a social media platform, media that captures interesting events is shared more frequently. The recording of predators with prey items falls into this category and represents a potentially rich data resource. Australia, in particular, has high public engagement in social media wildlife documentation, with many birdwatching enthusiasts posting media to Facebook and Instagram. For example, over 280,000 people are members of the Australian Native Birds Facebook group, representing over 1% of Australia's population and publishing over 3000 posts per month (Australian Native Birds (ANB) 2024). The scale of social media and the high quantity and quality of media posted offer the potential to be a single source for researchers, simplifying this method and maximising efficiency. In addition, traditional citizen science data from databases such as eBird have large potential errors and biases from observer variability (Gorleri et al. 2023). Mining social media posts, although potentially more time consuming, bypasses this issue by putting the onus of species identification accuracy on the researcher, the person most likely to be accurate. Also, social media companies such as Meta have been developing open‐access tools to aid researchers, improving search completeness and efficiency (Meta Platforms Inc. 2025). Finally, this source has proven transferable to other taxa and study areas. Examples include the use of social media images to determine trophic interactions of reptiles and amphibians (Maritz and Maritz 2020), and behaviour observations between coyotes ( *Canis latrans* ) and domestic dogs ( *C. lupus familiaris* ) (Boydston et al. 2018).

Two Australian diurnal raptors were chosen for the study; the Eastern Osprey ( *Pandion haliaetus* *cristatus*) and the White‐bellied Sea‐Eagle ( *Haliaeetus leucogaster* ) (WBSE hereafter). Primarily, these species were selected as they are located predominantly around Australia's coastline (Debus 2019). Australia's human population mirrors this distribution, with 87% of people residing within 50 km of the coast (Clark et al. 2021). This pattern reduces the impact from spatial sampling bias, where more citizen science observations occur in areas of greater human density and accessibility (Mair and Ruete 2016). This allowed other biases to be identified more easily. Secondly, although these species are listed as of Least Concern by the IUCN (IUCN 2025), they are good proxies for threatened raptor species who are photographed less frequently, providing a sample large enough to compare to existing diets and identify biases.

Previously quantified Eastern Osprey diets in Australia have been located in a small section of the country: the coast of northern New South Wales (NSW) (Clancy 2005) and at Lizard Island in Queensland (Smith 1985), making the diet unlikely to be representative of the total Australian distribution. In addition, the samples in both studies were exclusively taken from nest locations, indicating a temporal bias towards the breeding season. Most previously quantified diets of Australian WBSE were surprisingly based inland in the Australian Capital Territory (ACT) (Olsen et al. 2013) and NSW (Debus 2008), and at freshwater locations in the Northern Territory (NT) (Corbett and Hertog 2011a, 2011b), with a small coastal sample on Great Barrier Reef islands in QLD (Smith 1985). These diets indicated an opportunistic nature with greater take of locally abundant prey such as freshwater turtles in the NT and waterbirds in the ACT. This suggests that more quantified diet studies, particularly coastal, are required throughout the known distribution of Australian WBSEs to understand their diet. Furthermore, the absence of quantified diets for both Eastern Osprey and WBSE in Western Australia is of note. The secondary objective of this study is to expand the dietary knowledge of both raptor species, with diets sampled throughout their distribution, from many individuals in both breeding and non‐breeding seasons.

## 2 Materials and Methods

### 2.1 Data Acquisition From Social Media

Facebook and Instagram were selected as the social media platforms for the study based on their large quantity of relevant results, adequate searchability and repeatability, high quality media, and good availability of metadata. YouTube, Reddit, and TikTok were excluded due to small relevant sample sizes. X (formerly Twitter) was excluded due to a high occurrence of false positives when searching (i.e., unrelated content that returns on the same keywords). In this study, Facebook and Instagram were searched manually. However, it is noted that since the data collection in this study was completed, Meta has introduced the Content Library Application Programming Interface (API) which allows researchers to efficiently conduct repeatable searches of both platforms in their entirety and index up to 100,000 results (Meta Platforms Inc. 2025). For future researchers, using the Meta API for similar work should return more results, optimise time efficiency, and minimise duplication. This does not invalidate the method of this study, but highlights the fast‐developing nature and potential of using social media in citizen science methodologies.

Keywords (“raptor”, “prey”, “osprey”, “eagle”, “sea‐eagle”, “WBSE”) were searched in four Australian national and seven state or territory‐based Facebook groups. National groups included two raptor‐specific and two general birding groups. All state and territory‐based groups were for general birding. Birding groups were selected based on greatest membership and post frequency (Table A1). The desktop web browser version of Facebook was used for all searches. Group posts were filtered by year to reduce the processing power required to view each search. All keyword search returns per year and group were recorded in a single sitting so as not to refresh the Facebook search algorithm. This approach was checked by repeating some searches and appeared to produce repeatable results. However, some additional posts were found in searches performed at a substantially later date. This suggests the method is not completely repeatable and should be regarded as similar to a survey. Effort was made not to record duplicate posts when searching different keywords. However, some duplicate posts in and between different Facebook groups were recorded and subsequently excluded when the data were merged into a complete dataset. To ensure search consistency, LB completed all Eastern Osprey and WBSE searches in the national groups, MA all Eastern Osprey searches and HH all WBSE searches in the state groups.

Instagram posts were searched using keywords and specific hashtags (Table A2) on the phone application search tab. The Instagram desktop application and website were not used as they returned substantially fewer results. Each post recorded was favourited in the app to make it easier to identify duplicates between search terms and allow data recorders to pause searching as required. This was important as the Instagram search algorithm returns refresh each time. A repeat search of each keyword and hashtag was completed to maximise results. AR completed all Eastern Osprey searches and HH all WBSE searches on Instagram.

**FIGURE 1:**
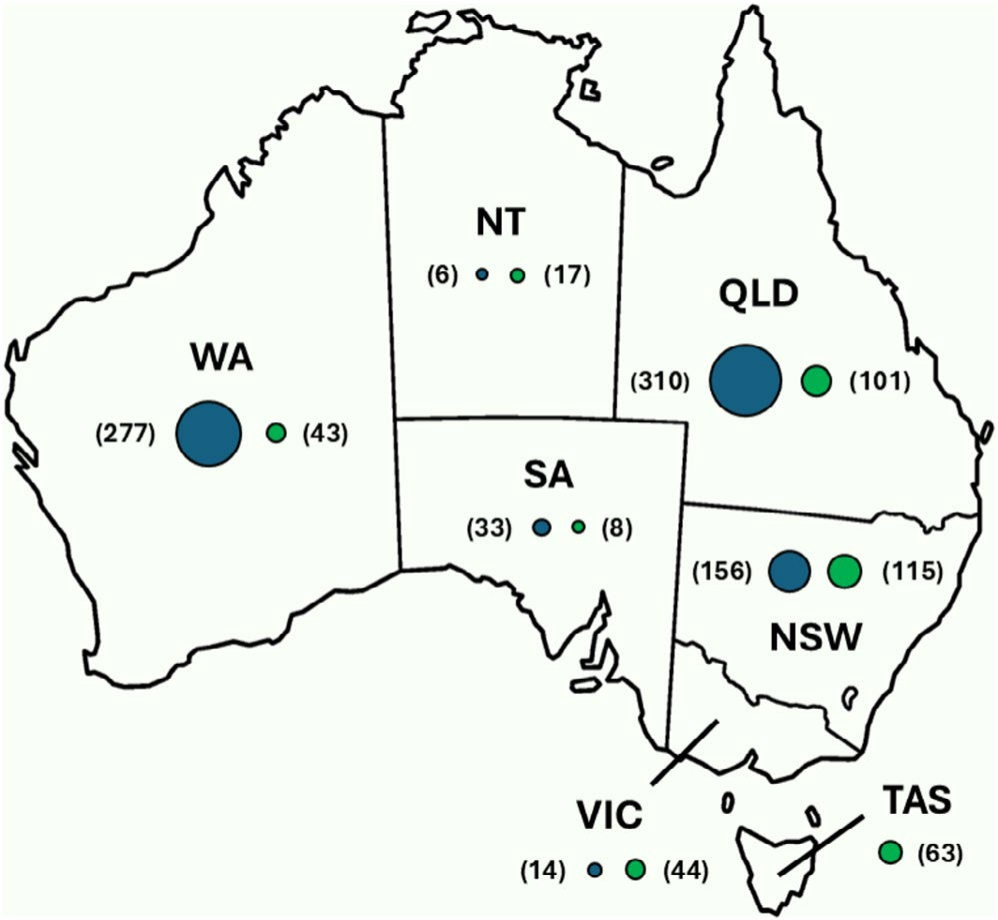
Distribution of social media posts (N) of Eastern Osprey (*Pandion haliaetus cristatus*) (blue) and White‐bellied Sea‐Eagle ( *Haliaeetus leucogaster* ) (green) in Australia.

Photographs and videos were stored for posts that satisfied the following inclusion criteria: target species in possession of a prey item, media recorded in Australia, posts dated between 1st January 2019 and 31st December 2023, and the raptor was not captive. This time period was selected due to many of the Facebook groups being formed at the beginning of this period or in the years just prior. When available, different views of the prey were recorded to assist identification. Metadata were recorded for each post: author, date posted, location (Australian state) (Figure 1), raptor species, and any prey identification by the public.

Duplicates for each platform were found using a multi‐stage process. Firstly, stored images were analysed for duplication using the AllDup duplicate file finding software (MTSD 2022). Secondly, duplicates were identified using the author name and date, checking content to confirm. Thirdly, potential duplicates were identified during the prey identification workshops and later confirmed. Finally, prey identification was used alongside the author name to identify duplicates; this was particularly useful for prey items that had been posted by the same author at significantly different time periods. All duplicates were marked in the index of prey items, and the earliest posted duplicate was retained in all cases. Where additional metadata was provided in duplicate posts, this was added to the original indexed prey item.

### 2.2 Prey Identification

Identification of prey items was not confirmed during the data collection process. Instead, several prey identification workshops, attended by a minimum of four authors, were completed. Each prey item was identified to the lowest possible taxonomic level based on physically identifiable features. Experts in the authorship team were consulted to validate identifications at the family level: NF for Macropodidae, GB and SD for Avian families. In addition, an external expert was consulted to validate the Osteichthyes families identified. The remaining prey families were confidently identified by the team and did not require further validation. Time recording was completed by all participants of the data collection and prey identification processes. The total time of four workshop participants plus expert validation (when applicable) was used for calculating the time taken during the prey identification process.

### 2.3 Imputation of WBSE Prey Family Identification

A strong bias in WBSE prey identification was found for unidentified Osteichthyes (52%) compared to other animal classes (0% to 17%). This represented a case of missing at random (MAR) categorical data, i.e., not randomly distributed, but accounted for by other observed variables. MAR data should undergo imputation to mitigate bias in subsequent analyses (Newman 2014), and multiple imputations were completed using the k nearest neighbour (kNN) function of the VIM package in RStudio (Kowarik and Templ 2016). kNN imputation is an appropriate and accurate method for MAR categorical data (Memon et al. 2023).

Multiple permutations of distance variables (existing prey family identifications, state/territory, coastal location, social media platform) were run while varying the *k* value (3, 5, 7, 9, 15). The imputations were compared to the existing prey identifications of each state and territory to estimate accuracy and identify anything unusual, for example overidentification of species or heavily reduced species richness. The best imputation was achieved by splitting the data by animal class and coastal location, removing the possibility of prey families being selected outside of their class or habitat (e.g., exclusively marine species). Imputations were then completed using existing prey family identifications and state/territory as the distance variables, with a *k* value of 5. Use of the social media platform variable over‐fitted the model and was excluded. A function setting was selected to exclude imputations from being used to impute further data. All other settings were left at the defaults detailed in the VIM package documentation (Templ et al. 2022).

Imputation was completed 100 times for each class with missing prey identification (Aves, Mammalia, Osteichthyes). Prey items with missing coastal location data (*n* = 33) were excluded. A total of 387 prey items were included in the process, and 154 (39.7%) prey items were imputed. Prey family identification was selected at random from the 100 imputations. Selection by median and modal values were trialled but was found to excessively bias prey identification to families with larger initial samples. Imputation was not required for Eastern Osprey as only the Osteichthyes component of the diets was compared due to the dominance of this animal class.

### 2.4 Comparison of Diets

The similarity of raptor diets in this study was investigated by: Australian state and territory, social media platform, and against previously published diets. Previously published WBSE diets differed by location, with inland diets being markedly different from coastal. Therefore, an additional comparison of coastal location was made for WBSE diets, with a distance to coastline of less than 5 km and/or the presence of shoreline or estuarine features considered coastal. All comparisons were made at the prey family level and completed using the built‐in functions of RStudio (Posit team 2024). Analyses of categorical data in contingency tables with small samples that are not well approximated by “named” distributions, such as chi‐square or normal, are completed using exact methods (Agresti 2013; Bilder and Loughin 2024). To allow for exact computation of larger I × J contingency tables, random samples from the tables with the given margins can be completed by Monte Carlo (Agresti 2013). In this study, two‐sided simulated probability values were calculated from Fisher's Exact tests for independence using Monte Carlo analyses run 20,000 times. A minimum total diet population cut‐off of 30 prey items was used during comparisons.

### 2.5 Comparison of Efficiency

Three efficiency metrics with the unit of hours per prey item were devised: data collection rate, prey identification rate, and these combined. Data collection rates were calculated for each raptor species and platform source (Facebook national groups, Facebook state/territory groups, Instagram) to identify any potential variance (*n* = 3 per species). Prey identification rates were recorded for the six prey identification workshops: Eastern Osprey (*n* = 4) and WBSE (*n* = 2). Expert validation time for prey items was added to prey identification time based on the quantity of prey identified for each raptor species. Combined rates were created based on the possible permutations for Eastern Osprey (*n* = 12) and WBSE (*n* = 6).

Efficiency of the social media method was compared against literature values for direct visual observations (Table A3) and nest cameras (Table A4). Direct visual observation identified prey during observations and was comparable to combined rates. Diet studies using nest cameras provided time estimations of how long cameras were active, not the human effort of recording observations, and were not comparable to this study. However, two of the nest camera articles did provide time estimates for reviewing video footage (Lewis, Fuller, et al. 2004; Steen 2009), with an average value of approximately 5% of total recording time. Prey identification rates for the four other nest camera studies were calculated using this average. These six values were compared to the prey identification rates from this study. Time efficiency data for pellet and remains collection, stable isotope analysis, and DNA metabarcoding methods were not available. Comparisons were completed using Welch's *t*‐tests (unequal sample size and variance).

## 3 Results

### 3.1 Diet Recorded, Eastern Osprey

A total of 1803 prey items for identification was stored, of which 1484 were included in subsequent analyses. Exclusions are detailed in Table A6. Of the 1452 prey items where animal class could be identified, 99.4% were Osteichthyes (bony fish). In addition, there were seven Chondrichthyes (cartilaginous fish); two from the Batoidea (ray), Glaucostegidae (shovelnose ray) and Triakidae (houndshark) families, and a single unknown shark species. Furthermore, a single Salvin's Prion ( *Pachyptila salvini* ) and a species of Majidae (spider crab) were recorded. In total, 879 prey items were identified from 48 families, 43 of which were Osteichthyes. Prey were identified at a rate of 97.8% by class, 59.2% by family, and 27.6% by species.

Facebook and Instagram produced Osteichthyes diets for Eastern Osprey that were likely to be similar in NSW (*p* = 0.739) and Western Australia (WA) (*p* = 0.132), but different in QLD (*p* = 0.003). Further inspection of the QLD diet revealed the difference was caused by inflated numbers of Siganidae (Happy Moments) and Sillaginidae (whiting), with 28 entries posted by seven specific authors, possibly causing a location bias. The remaining states and territories were excluded due to small sample size (< 30). Therefore, it was assumed that the platforms produced similar Eastern Osprey diets and were combined for subsequent analyses.

The number of prey items recorded each month during the cooler half of the year (May to October) was higher at 29 items compared to 20 prey items per month in the hotter months (November to April). Observations were lowest during March and April and highest during September and October. Total observations per year increased from their lowest of 244 in 2019 to their highest of 355 in 2021 before stabilising in subsequent years.

Location comparisons between NSW, QLD, South Australia (SA), and WA all yielded significantly different Eastern Osprey diets (*p* < 0.001, except SA vs. WA where *p* = 0.042). Comparisons with the NT and Victoria (VIC) were excluded due to sample size, and the Eastern Osprey range does not extend to Tasmania (TAS). Location differences in prey families are shown in the Osteichthyes component of Eastern Osprey diet recorded (Table A9).

A comparison was made between the NSW Osteichthyes diet from this study (*n* = 156) and an amalgamated diet (*n* = 104) from direct visual observations presented in three previous studies (Table A7). This comparison produced a significantly different diet (*p* = 0.006), with the primary difference observed in the Kyphosidae (Luderick) family, accounting for 14.1% of the Eastern Osprey diet compared to 2.9% previously. The diets were statistically similar when Luderick were excluded (*p* = 0.205).

### 3.2 Diet Recorded, White‐Bellied Sea‐Eagle

A total of 670 prey items for identification was stored, of which 501 were included in subsequent analyses. Exclusions are detailed in Table A6. A total of 466 prey items was identified from seven animal classes: Osteichthyes (71.0%), Aves (15.9%), Mammalia (6.4%), Reptilia (5.6%), Crustacea (0.4%), Mollusca (0.4%), and Chondrichthyes (0.2%). Of these prey items, a total of 284 prey items was identified from 55 families. Prey were identified at a rate of 93.0% by class, 56.7% by family, and 28.5% by species.

When comparing platforms, sample sizes were only large enough to compare Facebook and Instagram in the NSW coastal WBSE diet. This comparison indicated similarity between the platforms (*p* = 0.424). In addition, the QLD coastal WBSE diet was very close to the cut‐off (47 Facebook vs. 28 Instagram) and exhibited a very high probability of similarity between the two platforms (*p* = 0.983). Therefore, it was assumed that the platforms produced similar WBSE diets and were combined for subsequent analyses.

The number of prey items recorded each month during the cooler half of the year (May to October) was higher at 10 items compared to seven prey items per month in the hotter months (November to April). Observations were lowest in February and highest in June and August. Total observations per year increased from their lowest of 52 in 2019 to their highest of 126 in 2023.

Sample sizes were not large enough to compare any coastal and inland WBSE diets for any of the states and territories. However, the samples near the cut‐off indicated a difference (QLD *p* < 0.001, TAS *p* = 0.016), and although not proven conclusively, it is likely that coastal and inland WBSE diets are different and should be presented separately.

The coastal WBSE diets for NSW, QLD, TAS, and VIC were different (*p* < 0.001). In these diets, WBSE exhibited generalist prey selection with no family accounting for over 20% of the diet. Other coastal WBSE diets (NT, SA and WA) and all inland WBSE diets were below the cut‐off. To reflect the location differences, WBSE diet (excluding imputation data) has been presented for coastal and inland locations, and by state and territory (Table A10).

The coastal QLD WBSE diet from this study was significantly different (*p* < 0.001) from a diet published by Smith (1985) (Table A8). Previously published inland WBSE diets from the Australian Capital Territory (ACT), NSW, and the NT could not be compared due to the small inland sample sizes in our study.

### 3.3 Method Efficiency

In this study, 246 person‐hours were spent on data collection (searching, media download, recording metadata, duplicate removal) and 157 h of prey identification (identification workshops, expert validation). Dividing time spent on these activities by the total number of prey items provided efficiency in hours per prey item (Table 1). Both data collection and prey identification were faster for Eastern Osprey than WBSE.

**TABLE 1:** Efficiency of data collection and prey identification for the Eastern Osprey (*Pandion haliaetus cristatus*) and White‐bellied Sea‐Eagle ( *Haliaeetus leucogaster* ) diets sourced from social media in Australia.

Task	Eastern Osprey	WBSE
	Hr/prey	Hr/prey
Data collection	0.10	0.18
Prey identification	0.07	0.11
Total	0.17	0.29

Substantially more prey items were recorded from Facebook (*n* = 1386) than Instagram (*n* = 599) (Table 2). Despite this imbalance, there was no significant difference in data collection efficiency. Prey identification efficiency could not be compared between platforms as it was completed in unison. The prey identification rate to the family level was greater using media downloaded from Facebook.

**TABLE 2:** Comparison of using Facebook and Instagram platforms to obtain the diet of Eastern Osprey (*Pandion haliaetus cristatus*) and White‐bellied Sea‐Eagle ( *Haliaeetus leucogaster* ) in Australia.

Social media platform	Eastern Osprey prey	WBSE prey	Data collection efficiency	Prey identification rate	
	No.	No.	Hr/prey	Class	Family
Facebook	991	395	0.11	96.5%	60.6%
Instagram	493	106	0.10	96.8%	52.5%

The combined time efficiency (data collection and prey identification) of the social media method was significantly different from the literature values for direct visual observations (*t* = −2.5818, df = 10, *p* = 0.027), with a mean efficiency value over 25 times faster (Table A5). There was high variability in the literature efficiency values compared to the social media method. The prey identification efficiency of the social media method was not significantly different from the use of nest cameras (*t* = −1.61, df = 5, *p* = 0.168). Although the mean efficiency value was an order of magnitude less in the social media method, again there was high variability in the literature values, making the statistical test inconclusive.

## 4 Discussion

### 4.1 Diet Construction

The social media method produced large quantities of data, with prey totals for both case‐study species greater than or equivalent to previously published diets. Diets were recorded in regions of Australia where they were previously unknown. For example, a large Eastern Osprey diet profile was produced in WA (*n* = 227), showing a different Osteichthyes family composition from that of any other state. In addition, quantified WBSE diets were produced in TAS, VIC, and WA for the first time. This new diet information will be useful in producing future conservation strategies.

A small difference in WBSE diet was observed within 5 km of the coastline. However, this could not be proven statistically as all inland state and territory diets were too small to compare with their coastal equivalents. Previous WBSE diets were highly variable, with greater inland diet contributions from birds and mammals in the ACT and NSW (Debus 2008; Olsen et al. 2013, 2006), and reptiles in the NT (Corbett and Hertog 2011a), and a greater proportion of fish in coastal QLD (Smith 1985). Mostly, we did not observe the same high variability, with Osteichthyes consistently being the largest family class in all coastal and five out of seven inland diets, accounting for 66% of all prey items recorded. This finding suggests that previous localised diets are not representative of Australia‐wide WBSE diet and could reflect region‐specific prey availability or data collection bias from the method used.

In addition, we found some potential dietary specialisation in the WBSE diet. Elapidae, specifically sea snakes, accounted for over 13% of the QLD coastal diet. Although this result could be an identification bias (see Section 4.6), it could also reflect dietary preference in this region. WBSE were also recorded preying on Diodontidae (porcupinefish) and Tetraodontidae (toadfish) a total of 23 times, with 83% of these observations recorded in the south‐eastern states of NSW, TAS, and VIC (8.6%, 6.7%, and 8.5% of diets). This finding was unexpected, although both families were suspected to be in the diet (Smith 1985). To put this into context, the combined observations from these two families were greater than any other WBSE prey family recorded in this study. Porcupinefish and toadfish possess high levels of potent neurotoxins in their skin (Halstead 1965), indicating that WBSE can selectively dissect them or have evolved a resistance to their toxins.

### 4.2 Literature Comparison

The social media method produced a diet that was significantly different from observational diets published for Eastern Osprey in NSW (Clancy 1989, 2005; Kennard and Kennard 2006). However, the Osteichthyes family composition would have been similar without the large quantity of Luderick recorded in this study. Previous diets were recorded between 1980 and 2005, when Luderick was more frequently fished commercially in NSW. Annual catch was recorded as 580 t from 1991 to 1992 (Wilkinson 1997), compared with 248 t from 2019 to 2022 (Tuynman et al. 2023). This reduction in human consumption may explain the increase observed as fish populations rebound. In addition, Luderick are easy to identify with vertical stripes on the side of their body, and this identification bias may have increased recordings.

This study also recorded a WBSE diet that was significantly different from the diet recorded on several Great Barrier Reef islands, over 25 km from the mainland (Smith 1985). 80% of that reef diet was represented by just three prey families (Belonidae, Labridae, Procellariidae) found locally in high abundance. In our study, these families accounted for just 4% of the coastal QLD diet. This highlights the opportunistic nature of WBSEs and how they will adjust their diet according to local prey availability. Furthermore, our study had greater prey diversity in coastal QLD (*n* = 75, 22 prey families) compared to Smith's study (*n* = 79, 10 prey families), suggesting greater diet completeness in our study. In addition, Smith collected diet from prey remains, a method that has overestimated birds and large prey items in other large raptor diets (Lewis, Fuller, et al. 2004; Margalida et al. 2007). Procellariidae (shearwater) are seabirds, and Belonidae (longtom) and Labridae (wrasse) are fish families that commonly produce large specimens in Australia. Therefore, the same bias may have been present in the Smith (1985) study.

### 4.3 Prey Identification

**FIGURE 2:**
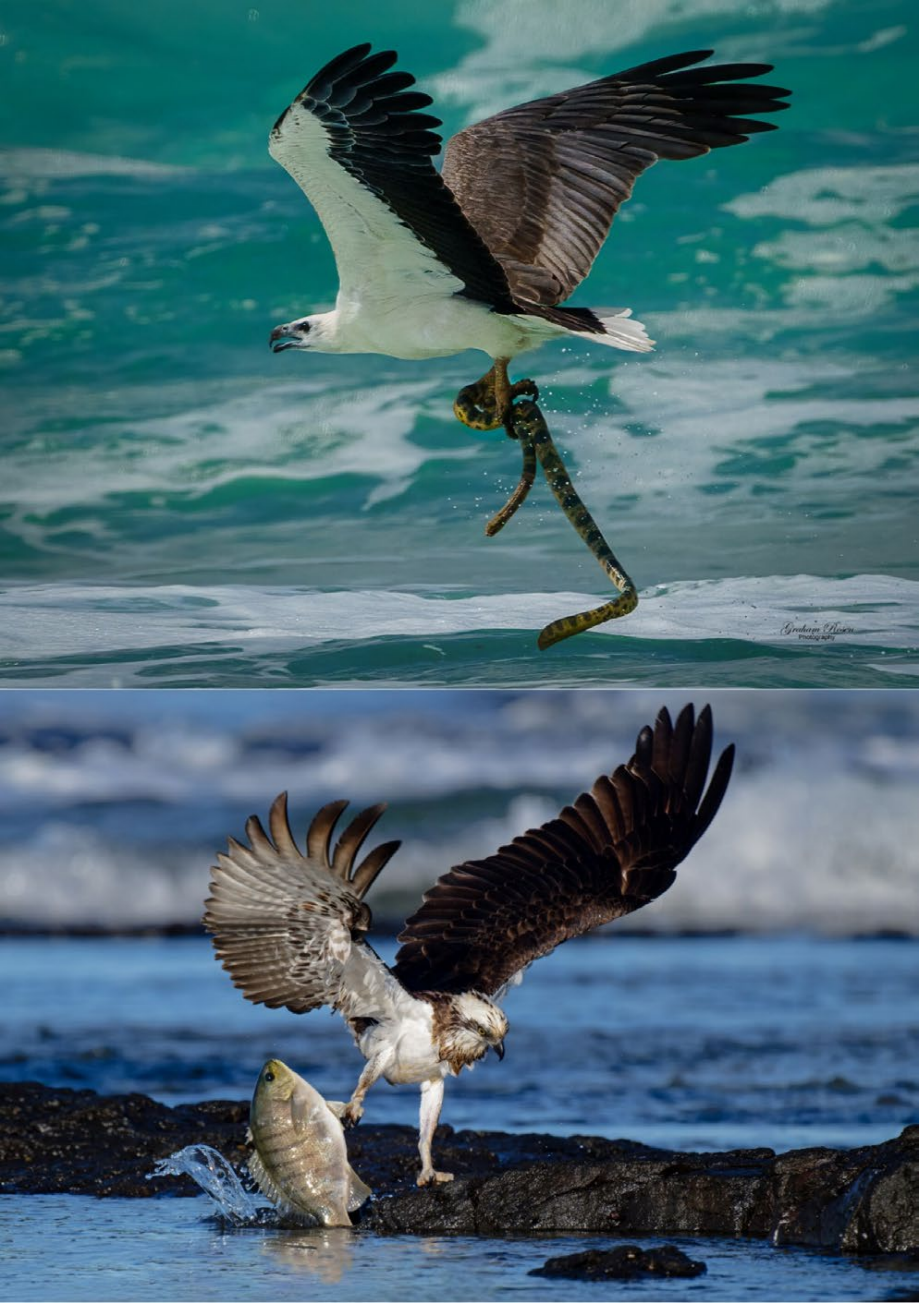
Examples of high quality raptor prey images from social media. Top: White‐bellied Sea‐Eagle ( *Haliaeetus leucogaster* ) with unidentified Elapidae species of sea snake at K'gari (Fraser Island) Beach, QLD (Rosen 2020); Bottom: Eastern Osprey ( *Pandion haliaetus* *cristatus*) with Luderick ( *Girella tricuspidata* ) at Flat Rock Beach, NSW (Casey 2023).

Many of the images posted to social media were of exceptional quality (Figure 2). However, species‐level prey identification was limited to 28%, as it was often not possible to see the features required, particularly with fish, of which Australia has over 4400 species (Australian Museum 2023). Family level prey identification of 59% for Eastern Osprey was greater than previous Australian studies using direct visual observations: 42% in Clancy (1989) and 19% in Kennard and Kennard (2006). It was also greater than the rate of 44% observed in the Canary Islands (Siverio et al. 2011). The corresponding rate for WBSE from this study was 57%, but we did not find any comparable studies that used direct visual observations. However, the species‐level prey identification rate in this study of 28% was substantially lower than Osprey and WBSE diets recorded using pellets, remains, or nest cameras (> 90%) (Cartron and Molles 2002; Corbett and Hertog 2011a; Glass and Watts 2009; Olsen et al. 2013; Siverio et al. 2011). Importantly, this study identified 879 Eastern Osprey prey items to family level, which is greater than the 729 prey items quantified in all four previous Australian studies. Combined with the quantification of diets for both raptors throughout their Australian distribution, the secondary objective of this study was achieved.

### 4.4 Method Efficiency, Data Collection and Prey Identification

The time efficiency of collecting and identifying prey items for Eastern Osprey was faster than WBSE. One reason for this difference was that most Eastern Osprey search results came from the ‘osprey’ search term, allowing for quick storage. WBSE searches took longer, as results came from a variety of search terms such as ‘eagle’, ‘sea‐eagle’, and ‘WBSE’. In addition, Eastern Osprey prey was nearly exclusively Osteichthyes, whereas WBSE prey also included birds, mammals and reptiles. Prey items were not sorted by animal class prior to the identification workshops, and as a result, Eastern Osprey prey identification ran more efficiently with only the single class to search in reference materials.

The time efficiency of collecting and identifying prey items using social media was significantly faster than the traditional method of direct visual observations. This difference was unsurprising, as the latency time between observations was absorbed by the social media author and was not accounted for. The time efficiency values from literature varied greatly, with raptors that capture smaller prey doing so more frequently to sustain themselves. For example, researchers observing Red‐footed Falcons ( *Falco vespertinus* ) that predominantly consume insects recorded a prey item every 0.1 h (Chavko and Krištín 2017), whereas for Golden Eagles ( *Aquila chrysaetos* ) that frequently preyed upon large mammals and snakes that take longer to consume, prey was recorded every 16.1 h (Seguin et al. 1998). The 0.2 h per prey item recorded in this study compares favourably, especially as Eastern Osprey and WBSE often consume large prey items (> 1 kg) and may only feed once or twice per day.

Prey identification efficiency of the social media method versus use of nest cameras was not significantly different. Both methods reviewed photographs and video footage for prey identification. However, the stored media in the social media method was on prey, whereas researchers needed to search through nest camera media to look for relevant incidents. This difference was reflected in the faster mean prey identification efficiency value of the social media method.

Unfortunately, the authors could not find any information on the time taken to collect and identify raptor prey from pellets, remains, stable isotope analysis, or DNA metabarcoding. Hence, it was not possible to directly compare the efficiency of these methods.

### 4.5 Social Media Platforms

Data collection efficiency from Facebook and Instagram was similar. However, Facebook had a greater prey family identification rate than Instagram. This may have been influenced by how media was stored from each platform; Facebook allowed direct download of images, whereas Instagram did not, and we were reliant on phone screenshots of lower quality. However, video footage from Facebook was typically low quality as historical videos are archived and compressed to save space on their servers.

Searching of Facebook groups had a good level of repeatability, with searches by year returning similar results each time if search terms were included in post descriptions or comments. The Instagram search function was less transparent, with posts that had greater interactions prioritised in returns. It was unclear if searches were actually complete or whether unpopular posts had been omitted. However, searching specific hashtags (i.e., #easternosprey) was more reliable, and Instagram did have a useful post favouriting option, helping to reduce duplication. Provision of metadata was similar between platforms, with most posts providing location and many providing a good attempt at prey identification.

The biggest difference between platforms was the quantity of prey items stored, with over twice as many stored from Facebook. This aspect suggests a greater number of members posting relevant material, although this may change over time as public demographic engagement with each platform changes. Recent platform usage figures from the UK showed that more people under 25 years of age use Instagram than Facebook, whereas the reverse is true for all other age categories (Smith 2024). This pattern may indicate a future shift to Instagram. However, the release of the Meta Content Library API may negate this. This research tool can search and index both platforms in their entirety and allows for faster, more complete, and repeatable searches of media posted on these platforms. Furthermore, it is anticipated that future development will incorporate Meta Artificial Intelligence into the API for more advanced queries. The authors recommend using this API for similar future research.

### 4.6 Method Bias and Prey Identification

Diets formulated from prey remains overestimate the presence of birds and large mammals; pellets can overestimate smaller mammals, and direct observations produce the most accurate diet (Lewis, Fuller, et al. 2004; Margalida et al. 2007). The NSW Eastern Osprey diet in this study was similar to previous NSW studies that used direct visual observations, with the main difference possibly from long‐term temporal changes in Kyphosidae (Luderick) populations. This outcome suggests that the social media method may have a similar level of prey identification bias. However, the increase in Luderick could also be because it was easily identifiable, and it is likely there was an identification bias in this study towards such prey. Other easily identified Osteichthyes families found in large quantities included Happy Moments, longtoms, porcupinefish, toadfish, Anguillidae (freshwater eels), Hemiramphidae (garfish), Monacanthidae (leatherjackets), and Rhombosoleidae (flounders). Collectively with Luderick, these families accounted for 16% of Eastern Osprey and 23% of WBSE prey. This proportion was higher than expected, with previous diets listing these families as minor or not at all.

It is also likely that more ‘exciting’ prey items are posted more frequently than is reflective of actual diet. For example, sea snakes accounting for 13% of the coastal QLD WBSE diet seems inflated compared with 4% listed by Smith (1985) on several Great Barrier Reef islands, an area with high numbers of sea snakes. Conversely, ‘boring’ prey items such as Mugilidae (mullets), or harder to identify species may have been underestimated.

The citizen science raptor diet study by Naude et al. (2019) also highlighted a potential bias to larger prey items as they are easier for bird photographers to spot and focus on, and smaller prey items are consumed more quickly. Eastern Osprey and WBSE typically eat fairly large prey items, and this bias may be limited here, but could be significant for other raptors. However, a study of Montagu's harriers by Kannan et al. (2023) found that photographs captured a higher proportion of insects in the diet than pellets. Furthermore, unpublished social media diet data from this study's authors of smaller raptors in Australia showed a high prevalence of small prey items at high resolution. However, difficulties in prey identification were apparent in all prey sizes if distinguishing features were not visible.

Bias was observed in prey family identification, with Osteichthyes prey more likely to remain unidentified than other animal classes. This problem was because many fish did not have enough identifying features visible in the media stored. To correct this bias, we imputed unidentified WBSE prey items to allow comparison of diets, and a similar approach may be required in future studies that vary by class. Researchers identified prey themselves rather than taking information from citizen science databases; thus, known biases in unreliable sampling and prey identifications were avoided.

### 4.7 Method Bias, Spatial

This method is capable of quickly collecting large quantities of prey items over a continental scale and removes sampling bias caused by studying a small percentage of a raptor population over a restricted spatial area, a bias imposed by the time and cost restrictions of other methods. However, several location biases were highlighted by this study. Firstly, we found instances where the same enthusiastic authors posted many times and, in some cases, posted many of the same prey species over a fairly localised area. This practice could cause a bias from high abundance of particular prey in the area they normally frequent. Similarly, there were certain birding hotspots where multiple birders liked to visit, such as the Western Treatment Plant in Werribee, which accounted for 29% of WBSE observations from VIC, and likely overcontributed locally common prey species such as waterbirds to the diet.

However, the largest and most significant location bias is towards areas of greatest human activity (Naude et al. 2019; Panter and Amar 2021; Panter et al. 2024). In simple terms, citizen science entries are heavily biased towards where humans spend their time and are more likely to observe wildlife. We chose two coastal raptors to mitigate this bias in Australia, as this is where most of the human population is located. This allowed us to reduce this bias and helped identify other biases. However, for non‐coastal species this bias needs to be quantified and the diet adjusted accordingly as suggested by Fithian et al. (2015).

### 4.8 Method Bias, Temporal

The social media method had less seasonal bias than other methods. Heat is a problem in Australia, with many wildlife surveys taking place in the cooler months of the year. Although there was an increase in observations during the cooler months (May to October), observations during the warmer months still accounted for 40% of all observations in this study. Other methods do not normally investigate seasonal bias due to time restraints, and many raptor diet studies are conducted during the breeding season, which aligns with the cooler months in Australia. This schedule is inherently biased, restricting knowledge of diets to a small portion of the year. The social media method has the benefit of being desktop based for researchers, reducing survey costs and avoiding unpleasant field conditions, and could be completed during the traditional quieter survey months.

However, daily temporal bias may also be present in this study, with most media captured during fair weather or the ‘golden hour’ to improve subject lighting. Many prey species change their behaviour during different periods of the day and in different weather. This pattern may result in increased proportions of certain species during the most frequently photographed conditions and could cause a significant bias in countries with greater variability in weather.

Despite the observation period including the COVID‐19 pandemic and associated lockdowns in 2020 and 2021, there was no significant difference in the annual number of prey items recorded for either raptor species. Total annual observations were similar to 2022 and 2023. In fact, 2019, prior to the pandemic, had the lowest number of observations. However, it should be noted that in Australia, walking was a permitted exemption from lockdown restrictions. As a result, COVID‐19 may have led to a boost in the number of people birding as people ventured outside and connected more with their local environments.

### 4.9 Limitations and Recommendations for Further Research

Duplicates were tricky and time‐consuming to identify and remove, and although we removed duplicates within social media platforms, a reliable method for identifying duplicates between Facebook and Instagram was not found. On Facebook, users normally use their name as their username, whereas on Instagram this is often not the case. Authors may post the same content on different days and, in some cases, years. Accurate location was not always posted. Therefore, there was no suitable metadata category that could be used. Available image analysis software packages that can identify duplicate images failed as the images from Instagram were stored via screenshot and could not be matched.

Another limitation was the previously described repeatability and transparency of searches with both platforms. Also, it was not possible to standardise keyword searches between the two platforms as Facebook groups were searched versus the entirety of Instagram. In addition, Facebook would shuffle search returns, and so reviews of each search term results had to be completed in one sitting. Long search returns on Facebook used substantial amounts of computer memory to load and, on occasion, would crash before the full list could be reviewed. Furthermore, older media is compressed by platforms to save server space, resulting in lower quality videos that were hard to use for prey identification. These issues should be avoided by using the Meta Content Library API for future studies.

We used two raptors that were fairly similar: large, fairly common, coastal, and mostly consume fish. It could be more difficult to accumulate diets of smaller, elusive, or inland raptor species. However, the authors have successfully used this method to record prey items for all Australian raptors (unpublished data). Another limitation was that all prey items were assumed to be consumed, whereas some prey may have been discarded, for example, the toxic porcupine fishes or toadfishes. It was also not always possible to know if the raptor hunted the prey or scavenged a carcass.

Finally, the lack of specific location data limited spatial observations in this study. Many posts did not provide an exact location, and the authors chose to record the Australian state as this was often provided or could be easily deduced. In hindsight, it would have been useful to record both, even if the exact location was largely incomplete. Future research using the Meta Content Library API may be able to extract location information more easily. Much of the spatial analysis in this study was limited to state‐based regions, whereas environmental or climatic factors could be investigated with precise location. Future social media diet studies should store the exact location and explore these factors.

Future research should also focus on quantifying the biases of this method for a mixture of raptor species in different countries. This focus will ensure that the method can be used to produce accurate diets for other raptors. Research on the time efficiency of collecting raptor diet from pellets, remains, stable isotope analysis, and DNA metabarcoding is also required to establish direct comparisons.

The method has potential for documenting the diet of other taxa. However, the accuracy, efficiency, and biases for other taxa would need to be established. This method could also be used for other biological monitoring. For example, age demographics could be monitored to provide breeding information and estimate mortality rates. Overall, the use of social media was an efficient method for collecting raptor diet information and has potential for expansion to other species and areas of research.

## Author Contributions

**Leo Biggs:** conceptualization (lead), data curation (lead), formal analysis (lead), investigation (lead), methodology (lead), project administration (lead), software (lead), supervision (lead), writing – original draft (lead), writing – review and editing (equal). **Greg S. Baxter:** conceptualization (supporting), data curation (supporting), investigation (supporting), methodology (supporting), supervision (supporting), validation (equal), writing – original draft (supporting), writing – review and editing (equal). **Stephen J. S. Debus:** conceptualization (supporting), data curation (supporting), investigation (supporting), methodology (supporting), supervision (supporting), validation (equal), writing – original draft (supporting), writing – review and editing (equal). **Neal Finch:** conceptualization (supporting), data curation (supporting), investigation (supporting), methodology (supporting), supervision (supporting), validation (equal), writing – original draft (supporting), writing – review and editing (equal). **Anysha Riggs:** data curation (supporting), investigation (supporting), writing – original draft (supporting), writing – review and editing (equal). **Hayden Houweling:** data curation (supporting), investigation (supporting), writing – original draft (supporting), writing – review and editing (equal). **Melissa Appleby:** data curation (supporting), investigation (supporting), writing – original draft (supporting), writing – review and editing (equal). **Peter J. Murray:** conceptualization (lead), data curation (supporting), formal analysis (supporting), investigation (supporting), methodology (lead), project administration (supporting), supervision (lead), validation (equal), writing – original draft (supporting), writing – review and editing (equal).

## Conflicts of Interest

The authors declare no conflicts of interest.

## Data Availability

All the required data are uploaded as Appendices.

## Appendix A

**TABLE A1:** Membership population of each birding group on Facebook on the 30th September 2024.

Facebook group	Acronym	Membership
Australian Native Birds	ANB	280,600
BirdLife Australia Raptor Group	BARG	4700
Bird Photography Australia	BPA	81,900
Birds of Prey (Australia & New Zealand)	BPOZ	3900
Birds NSW	NSWB	2200
Northern Territory Birders	NTB	6700
Brisbane Birds	QLDB	15,200
South Aussie Birding	SAB	7600
Tasmanian Bird Sightings and Photography	TASB	13,500
Victorian Birders	VICB	11,400
Western Australian Birds	WAB	32,600

**TABLE A2:** Hashtags and terms used to search Instagram for posts containing either Eastern Osprey (*Pandion haliaetus cristatus*) or White‐bellied Sea‐Eagle ( *Haliaeetus leucogaster* ) in possession of a prey item.

Eastern Osprey	White‐bellied Sea‐Eagle
#australianbirdsofprey	#australianbirdsofprey
#australianosprey	#australianeagle
#australianraptors	#australianraptors
#birdsofpreyaustralia	#birdsofpreyaustralia
#easternosprey	#eagleaustralia
#ospreyaustralia	#eaglesofaustralia
#ospreyofaustralia	#haliaeetusleucogaster
#ospreysofaustralia	#raptorsofaustralia
#pandioncristatus	#WBSE
#pandionhaliaetuscristatus	#whitebelliedeagle
#raptorsofaustralia	#whitebelliedseaeagle
eastern osprey	WBSE australia
easternosprey	WBSEaustralia
osprey australia	White bellied sea eagle australia
ospreyaustralia	Whitebelliedseaeagle australia
	Whitebelliedseaeagleaustralia

**TABLE A3:** Observation rates of raptor diet studies utilising the method of direct visual observation.

Author	Year	Raptor species	Observation	Prey items	Observation rate
			Hr	No.	Hr/prey
Bakaloudis et al.	2011	Long‐legged Buzzard *Buteo rufinus*	216.5	61	3.55
Cardador et al.	2012	Marsh Harrier *Circus aeruginosus*	1049	752	1.39
Chavko and Kristin	2017	Red‐footed Falcon *Falco vespertinus*	31.4	305	0.10
Dykstra et al.	2003	Red‐shouldered Hawk *Buteo lineatus*	300.0	249	1.20
Margalida et al.	2007	Bearded Vulture *Gypaetus barbatus*	2206	526	4.19
Pavez et al.	1992	Black‐chested Buzzard‐Eagle *Geranoaetus melanoleucus*	374	84	4.45
Real	1996	Bonelli's Eagle *Hieraaetus fasciatus*	681.7	124	5.50
Redpath et al.	2001	Hen Harrier *Circus cyaneus*	1540	1332	1.16
Schulze et al.	2000	Double‐toothed Kites *Harpagus bidentatus*	817	517	1.58
Seguin et al.	1998	Golden Eagle *Aquila chrysaetos*	1271	79	16.09
Zuluaga and Echeverry‐Galvis	2016	Black‐and‐chestnut Eagle *Spizaetus isidori*	440	21	20.95

**TABLE A4:** Prey Identification rates of raptor diet studies utilising the method of nest camera observation.

Author	Year	Raptor species	Prey identification	Prey	Identification rate
			Hr	No.	Hr/prey
Cava et al.	2012	Cooper's Hawks *Accipiter cooperii*	65^a^	783	0.08
Lewis et al.	2004	Northern Goshawks *Accipiter gentilis*	430	1540	0.28
McPherson et al.	2016	African Crowned Eagle *Stephanoaetus coronatus*	2046^a^	836	2.45
Steen	2009	Eurasian Kestrel *Falco tinnunculus*	130	3867	0.03
Steen et al.	2011	Eurasian Kestrel *Falco tinnunculus*	125^a^	2282	0.05
van der Meer et al.	2018	African Crowned Eagle *Stephanoaetus coronatus*	892^a^	509	1.75

^a^ Prey identification hours calculated from total recording hours of nest cameras and the average ratio (5.3%) of prey identification hours to recording hours of Lewis, Fuller, et al. (2004) and Steen (2009).

**TABLE A5:** Two‐sided Welch's *t*‐test results of different hypotheses comparing the efficiency of the social media method with traditional methods of direct visual observations and the use of nest cameras.

Comparison hypotheses	Social media method efficiency	Traditional method efficiency	Difference	*t*	*d*	*p*
Is collecting and identifying raptor diet prey items from social media more efficient than from direct visual observations?	*M* = 0.21 h/prey SD = 0.05 Range = 0.15–0.29	*M* = 5.47 h/prey SD = 6.44 Range = 0.10–20.95	5.26 h/prey SD = 2.04	*t*(10) = −2.58	−1.15	0.027
Is identifying raptor diet prey items from social media more efficient than from nest cameras?	*M* = 0.08 h/prey SD = 0.01 Range = 0.06–0.10	*M* = 0.78 h/prey SD = 0.96 Range = 0.03–2.45	0.70 h/prey SD = 0.43	*t*(5) = −1.61	−1.02	0.168

**TABLE A6:** Exclusion criteria for social media posts stored of Eastern Osprey (*Pandion haliaetus cristatus*) and White‐bellied Sea‐Eagle ( *Haliaeetus leucogaster* ) with prey items that were subsequently excluded from the study analyses.

Exclusion criteria	Eastern Osprey	White‐bellied Sea‐Eagle
Duplicate post	292	138
Not target raptor species/subspecies	11	8
Posted outside of study period	8	0
Prey item fed to raptor by human	4	15
Prey item not visible	2	6
Raptor not in possession of prey item	2	2
Total	319	169

**TABLE A7:** The diet recorded from direct visual observations of Eastern Osprey (*Pandion haliaetus cristatus*) in three studies from New South Wales, Australia.

Osteichthyes Family	Common name(s)	Clancy (1989)	Clancy (2005)	Kennard and Kennard (2006)
		*n* = 16	*n* = 38	*n* = 50
Alosidae	Pilchard			2.0%
Carangidae	Trevally, Yellow‐tail			14.0%
Hemiramphidae	Garfish		2.6%	
Kyphosidae	Luderick, Drummer		2.6%	4.0%
Moridae	Scorpionfish	6.3%		
Mugilidae	Mullet	56.3%	86.8%	10.0%
Platycephalidae	Flathead			2.0%
Pomatomidae	Tailor			2.0%
Scombridae	Mackerel			8.0%
Sillaginidae	Whiting	25.0%		18.0%
Soleidae	Sole		2.6%	
Sparidae	Bream, Tarwhine, Snapper	12.5%	2.6%	38.0%
Tetraodontidae	Toadfish			2.0%
Trichiuridae	Hairtail		2.6%	

**TABLE A8:** The diet recorded of White‐bellied Sea‐Eagle ( *Haliaeetus leucogaster* ) at Great Barrier Reef islands of Queensland, Australia.

Class	Family	Common name	Smith (1985)
			*n* = 79
Aves	Ardeidae	Heron	1
	Laridae	Tern	6
	Procellariidae	Shearwater	28
Osteichthyes	Belonidae	Longtom	26
	Carangidae	Scad	1
	Fistulariidae	Cornetfish	1
	Labridae	Wrasse	9
	Scaridae	Parrotfish	3
	Tetraodontidae	Toadfish	1
Reptilia	Elapidae	Sea snake	3

**TABLE A9:** Prey family contributions to the Osteichthyes component of Eastern Osprey (*Pandion haliaetus cristatus*) diet in Australia from this study.

Osteichthyes family	Common name(s)	NSW	NT	QLD	SA	VIC	WA
		*n* = 156	*n* = 6	*n* = 310	*n* = 33	*n* = 14	*n* = 227
Anguillidae	Freshwater eel			0.3%			
Ariidae	Catfish			0.3%			
Arripidae	Australian salmon, herring			0.3%	6.1%		0.9%
Belonidae	Longtom			1.6%			1.8%
Carangidae	Trevally, dart, scad, kingfish, queenfish, runner	5.8%	16.7%	7.7%	9.1%		0.4%
Chaetodontidae	Butterflyfish						0.4%
Chirocentridae	Wolf‐herring			0.3%			
Cichlidae	Tilapia			3.2%			
Clupeidae	Freshwater herring			1.9%			
Cyprinidae	Carp, goldfish			0.3%			0.4%
Diodontidae	Porcupinefish			0.3%			0.4%
Dorosomatidae	Bony bream			3.2%			
Elopidae	Giant herring						0.9%
Gadidae	Cod				3.0%		0.9%
Girellidae	Rock blackfish						0.9%
Haemulidae	Sweetlips						0.4%
Hemiramphidae	Garfish			2.3%			1.8%
Kyphosidae	Luderick	14.1%		0.6%		14.3%	0.4%
Labridae	Wrasse, tuskfish, groper	0.6%		0.3%			5.3%
Latidae	Barramundi			0.3%			
Lutjanidae	Snapper, jack			1.3%			
Megalopidae	Tarpon			0.3%			
Monacanthidae	Leatherjacket				3.0%		0.4%
Monodactylidae	Butter bream						
Mugilidae	Mullet	42.9%	33.3%	33.9%	39.4%	42.9%	53.3%
Odacidae	Cale						0.9%
Percichthyidae	Temperate perch			0.6%			
Platycephalidae	Flathead	1.3%		0.6%			0.9%
Plotosidae	Cobbler						0.4%
Pomacentridae	McCulloch's scalyfin						1.8%
Pomatomidae	Tailor						0.9%
Rhombosoleidae	Flounder			0.3%	15.2%		5.7%
Scaridae	Parrotfish			0.3%			1.3%
Scatophagidae	Scat		50.0%	1.6%			
Scombridae	Mackerel, bonito			1.0%			1.3%
Serranidae	Grouper, rockcod	0.6%					2.2%
Siganidae	Happy moment			11.6%			1.3%
Sillaginidae	Whiting	16.0%		8.4%	6.1%	14.3%	3.1%
Soleidae	Sole			0.3%			
Sparidae	Bream, tarwhine, snapper	17.3%		15.5%	6.1%	14.3%	7.9%
Sphyraenidae	Barracuda				3.0%		
Terapontidae	Trumpeter, perch			0.3%		14.3%	1.3%
Tetraodontidae	Toadfish	1.3%		0.6%	9.1%		2.2%

**TABLE A10:** Prey family contributions to the White‐bellied Sea‐Eagle ( *Haliaeetus leucogaster* ) diet in Australia from this study, split by state/territory and coastal (C) or inland (I) location.

Class	Prey family	Common name(s)	NSW		NT		QLD		SA		TAS		VIC		WA	
			C	I	C	I	C	I	C	I	C	I	C	I	C	I
			*n* = 108	*n* = 7	*n* = 1	*n* = 16	*n* = 75	*n* = 26	*n* = 5	*n* = 3	*n* = 43	*n* = 20	*n* = 34	*n* = 10	*n* = 28	*n* = 15
Aves	Anatidae	Duck, shelduck					1.3%			33.3%			8.8%	10.0%		20.0%
	Ardeidae	Heron														
	Charadriidae	Lapwing											2.9%			
	Columbidae	Dove														
	Corvidae	Corvid						3.8%								
	Laridae	Gull, tern	0.9%								9.3%				3.6%	
	Pelecanidae	Pelican														13.3%
	Phalacrocoracidae	Cormorant								33.3%			2.9%			
	Podicipedidae	Grebe													3.6%	
	Procellariidae	Shearwater	6.5%				2.7%				2.3%					
	Rallidae	Coot, nativehen, swamphen	0.9%	14.3%				11.5%				15.0%	17.6%	20.0%		6.7%
	Spheniscidae	Penguin	1.9%								4.7%		2.9%			
	Threskiornithidae	Ibis														6.7%
	Unknown	Aves sp.									2.3%	5.0%				
Chondrichthyes	Batoidea	Ray					1.3%									
Crustacea	Portunidae	Crab	0.9%				1.3%									
Mammalia	Bovidae	Cattle, sheep		14.3%								5.0%				6.7%
	Felidae	Feral cat													7.1%	
	Leporidae	Rabbit									2.3%	10.0%				
	Macropodidae	Kangaroo, pademelon, wallaby				6.3%	4.0%				4.7%	5.0%				
	Muridae	Rakali														
	Ornithorhynchidae	Platypus	0.9%													
	Pteropodidae	Flying fox													3.6%	
	Suidae	Feral pig				6.3%	1.3%	3.8%								
	Unknown	Mammal sp.					1.3%				2.3%	5.0%		10.0%	3.6%	
Mollusca	Sepiidae	Cuttlefish	0.9%													
	Unknown	Squid sp.									2.3%					
Osteichthyes	Anguillidae	Freshwater eel	0.9%	14.3%			1.3%	19.2%			9.3%	25.0%				
	Arripidae	Australian salmon	1.9%													
Osteichthyes	Belonidae	Longtom	3.7%			12.5%	1.3%									
	Carangidae	Dart, kingfish, queenfish					8.0%									
	Clupeidae	Freshwater herring						7.7%			2.3%	5.0%				
	Cyprinidae	Carp						3.8%		33.3%	2.3%					13.3%
	Dinolestidae	Pike														
	Diodontidae	Porcupinefish	2.8%				1.3%				9.3%	5.0%	5.9%			
	Kyphosidae	Luderick, rock blackfish	1.9%													
	Latidae	Barramundi				18.8%	1.3%									
	Monacanthidae	Leatherjacket	6.5%								4.7%		2.9%			
	Monodactylidae	Butter bream					1.3%									
	Mugilidae	Mullet	8.3%			6.3%	2.7%	3.8%			2.3%				7.1%	6.7%
	Percichthyidae	Bass, temperate perch	0.9%					3.8%								
	Percidae	Redfin perch										5.0%				
	Platycephalidae	Flathead					4.0%									
	Salmonidae	Salmon, trout									2.3%	5.0%		20.0%		
	Scaridae	Parrotfish	0.9%													
	Scatophagidae	Scat	2.8%				1.3%									
	Scombridae	Mackerel, tuna	1.9%		100.0%		2.7%									
	Scorpaenidae	Scorpaenidae sp.									2.3%					
	Siganidae	Happy moment					1.3%									

	Sillaginidae	Whiting	1.9%				1.3%								7.1%	
	Sparidae	Bream, snapper, tarwhine	6.5%				5.3%						14.7%			
	Terapontidae	Perch		14.3%												
	Tetraodontidae	Toadfish	6.5%			6.3%							5.9%			
Unknown	Osteichthyes sp.	37.0%	28.6%		25.0%	36.0%	38.5%	100.0%		34.9%	10.0%	35.3%	40.0%	50.0%	20.0%
Reptilia	Agamidae	Water dragon					1.3%									
	Chelidae	Freshwater turtle	0.9%	14.3%		18.8%		3.8%							3.6%	6.7%
	Cheloniidae	Marine turtle					2.7%									
	Elapidae	Sea snake	0.9%				13.3%								10.7%	
	Scincidae	Skink	0.9%													
